# A Brief History of the European Society for the Study of Tourette Syndrome

**DOI:** 10.3233/BEN-120287

**Published:** 2013

**Authors:** Hugh Rickards, Peristera Paschou, Renata Rizzo, Jeremy S. Stern

**Affiliations:** ^1^Department of NeuropsychiatryUniversity of BirminghamBirminghamUK; ^2^Department of Molecular Biology and GeneticsDemocritus University of ThraceThraceGreece; ^3^Department of Medical and Pediatric SciencesCatania UniversityCataniaItaly; ^4^Department of NeurologySt. George’s University of LondonLondonUK

**Keywords:** Tourette syndrome, tics, Europe, research, European Society for the Study of Tourette syndrome

## Abstract

The European Society for the Study of Tourette syndrome (ESSTS) was established in Denmark in 2000 by Mary Robertson and Anne Korsgaard. The aims of the organisation are to foster research activity and raise awareness of Tourette syndrome throughout Europe. The organisation went into abeyance in 2002 but was resurrected in 2007 in Bari, Italy. Since that time ESSTS has grown and prospered. We have established elected officers and a constitution. We have successfully applied for three large scale European research grants and have members throughout the European Union. We have held yearly meetings across Europe including two training schools and we have developed successful alliances with patient support groups. ESSTS has developed and published the first European guidelines on assessment, diagnosis and treatment of Tourette syndrome.

Tourette syndrome (TS) can be claimed by European neurologists as a condition with a local heritage (or perhaps by French neurologists as a Gallic condition, *par excellence*). The syndrome's early connotations as a bizarre and rare condition from the time of its 1885 case series description by Gilles de la Tourette in Paris lasted until well after the focus moved to New York in the 1970s when prevalence, rather than rarity, started to be observed. The systematic descriptive approach of Arthur and Elaine Shapiro at that time has continued, but even to this day has not allowed the condition to entirely shed its unusual lustre of Salpêtrière-associated exoticism amongst non-specialist clinicians. This has led to many patients not benefiting from appropriate management. Over a century after Gilles de la Tourette died from neurosyphilis he would be truly astonished to know that media interest in the condition has meant his name is widely known by the public in many parts of the world. This has brought both advantages and problems for affected families. The centre for the most committed progress in TS continued to be the USA, facilitated by the success of the Tourette Syndrome Association in targeted support of research. Alongside this explosion of the literature, since the 1980s there have also been significant contributions from Europe starting with the work of Mary Robertson and many of those who have worked with her.

The first meeting of ESSTS (European Society for Study of Tourette syndrome) was held in October 2000 in Copenhagen. Dr Anne Korsgaard, neurologist from Denmark, hosted a one day satellite symposium on TS which was attended by clinicians and researchers from a variety of disciplines. At that meeting, ESSTS was formed and jointly chaired by Anne Korsgaard and Mary Robertson. Initial committee members were Peter Heutink, Mauro Porta, Kirsten Mueller-Vahl and Finn Knudsen. The main aims of the association were to foster research activity and to raise awareness of TS around Europe.

Professor Robertson then began to gather names and details methodically of members of the association. This resulted in a meeting on the 20th September 2002

ISSN 0953-4180/13/$27.50 © 2013 – IOS Press and the authors. All rights reserved
This article is published online with Open Access and distributed under the terms of the Creative Commons Attribution Non-Commercial License at University College London. Attendees were from around Europe and all the speakers were from the UK on a variety of TS topics including discussions of the PANDAS hypothesis. At this meeting, the organisation settled on the name "ESSTS" and plans were made for further meetings.

For reasons of personal illness, the organisation became dormant at that stage. Very little occurred until a meeting in April 2007 of the Italian Movement Disorder group in Bari, Italy. At that meeting, the organisation was re-formed and the following year a meeting was held in Leiden when Hugh Rickards was formally elected Chair with Renata Rizzo secretary and Jeremy Stern treasurer. The first meeting of a group aiming to win an FP7 grant to study the link between streptococcal infection and TS was convened and plans commenced to submit for this.

The following year, 2009, the group met in Budapest. At this meeting, working groups were formed to look at the current evidence and best practice on diagnosis and treatment of TS and related conditions. The work was subsequently submitted and published last year as the first European guidelines on TS. This has been a lasting achievement of the group and the guidelines were recently the subject of a commentary in the British Medical Journal by Prof James Leckman. A website was set up and linked to patient support groups (http://tourette-eu.org).

In the meantime, leading members of ESSTS steered an application for a collaborative FP7-HEALTH project as well as an application for a COST Action for TS (European Cooperation in Science and Technology). The meeting in 2010 was hosted in Dresden by Veit Roessner. Here we learned that the COST application was successful and the COST Action "European Network for the Study of GTS" was established, chaired by Peristera Paschou. The COST Action (BM0905) has since led to vibrant meetings, workshops, scientific missions, and training schools for TS, aiming to promote knowledge, coordinate TS research around Europe and raise public awareness about the disorder (http://tourette-eu.org). In 2010, we re-shaped our constitution and set up some rules, which we published. Members were able to make short presentations of interesting cases and new data and we developed the idea of a video case workshop where people could present videos of interesting or difficult to diagnose cases. We were now able to get web-based feedback from meetings and we performed the first European survey of treatment practice via "Survey Monkey". For the first time, poster presentations made an appearance at the meeting.

In 2011, the meeting was held in London in partnership with the UK patient group Tourettes Action. A highly successful training school for psychological therapy in TS (particularly habit reversal) was organised by the COST Action, and hosted by Tourettes Action UK, in conjunction with the ESSTS Annual Meeting. At the 2011 Annual ESSTS Meeting, Hugh Rickards stood down as Chair and Peristera Paschou was elected to chair the organisation. Renata Rizzo was re-elected as Secretary and Pieter Hoekstra as Treasurer of ESSTS, while Kirsten Mueller-Vahl was elected ESSTS Vice-Chair. Members made brief presentations and the video case workshops were as popular as ever. The conference dinner was held in the Houses of Parliament as guests of an interested MP. It was at the 2011 Annual ESSTS Meeting that we learned of the success of our FP7-HEALTH application for EMTICS (European Multicentre Tics in Children Studies), coordinated by Pieter Hoekstra. EMTICS is an interdisciplinary project aiming to investigate the etiology of TS in a large patient cohort from around Europe, disentangling the relative contribution of environment and genetics in the development and course of the disorder.

The 2012 Annual ESSTS Meeting was held in Catania, Sicily, hosted by Renata Rizzo, and included a COST Action (BM0905) training school for TS clinical instruments and a meeting of the EMTICS Steering Committee in addition to the main ESSTS meeting and International Conference for Tourette Syndrome. Furthermore, with the support of COST Action BM0905, Tourette syndrome patient support and advocacy groups from around Europe came together in the "First International Meeting of Tourette Syndrome Support Groups", aiming to coordinate outreach activities across Europe.

Over the last five years, the group has grown in numbers and strength. Meetings devoted to TS have now become a normal occurrence and have been very successful in fostering a European community of clinicians and scientists. We have a robust constitution and have been successful at obtaining grant funding and collaborating around the EU. We have produced high quality education and training material and we have generated a genuine collegiate feeling between researchers. There are a number of challenges for the future. We need to maintain our momentum in more austere times. We need to make sure that we continue to keep focussed on the needs of patients and their families and to deliver today's best care, as well as providing hope for the future from research. European interest in TS is well and truly re-activated, and working together across our national boundaries can now powerfully accelerate progress. A lasting legacy of this organisation could be the formation and maintenance of a longitudinal database to produce the first genuine prospective data in TS. This would be a monumental achievement.
